# Copy number variation in bipolar disorder

**DOI:** 10.1038/mp.2014.174

**Published:** 2015-01-06

**Authors:** E K Green, E Rees, J T R Walters, K-G Smith, L Forty, D Grozeva, J L Moran, P Sklar, S Ripke, K D Chambert, G Genovese, S A McCarroll, I Jones, L Jones, M J Owen, M C O'Donovan, N Craddock, G Kirov

**Affiliations:** 1 School of Biomedical and Healthcare Sciences, Plymouth University Peninsula Schools of Medicine and Dentistry, Plymouth, UK; 2 MRC Centre for Neuropsychiatric Genetics and Genomics, Institute of Psychological Medicine and Clinical Neurosciences, Cardiff University, Cardiff, UK; 3 Department of Psychiatry, School of Clinical and Experimental Medicine, National Centre for Mental Health, University of Birmingham, Birmingham, UK; 4 Department of Medical Genetics, Cambridge Institute for Medical Research, University of Cambridge, Cambridge, UK; 5 Stanley Center for Psychiatric Research, The Broad Institute of MIT and Harvard, Cambridge, MA, USA; 6 Analytic and Translational Genetics Unit, Massachusetts General Hospital, Boston, MA, USA; 7 Division of Psychiatric Genomics in the Department of Psychiatry, Friedman Brain Institute, and Institute for Genomics and Multiscale Biology, Icahn School of Medicine, Mount Sinai, New York, NY, USA

## Abstract

Large (>100 kb), rare (<1% in the population) copy number variants (CNVs) have been shown to confer risk for schizophrenia (SZ), but the findings for bipolar disorder (BD) are less clear. In a new BD sample from the United Kingdom (*n*=2591), we have examined the occurrence of CNVs and compared this with previously reported samples of 6882 SZ and 8842 control subjects. When combined with previous data, we find evidence for a contribution to BD for three SZ-associated CNV loci: duplications at 1q21.1 (*P*=0.022), deletions at 3q29 (*P*=0.03) and duplications at 16p11.2 (*P*=2.3 × 10^−4^). The latter survives multiple-testing correction for the number of recurrent large CNV loci in the genome. Genes in 20 regions (total of 55 genes) were enriched for rare exonic CNVs among BD cases, but none of these survives correction for multiple testing. Finally, our data provide strong support for the hypothesis of a lesser contribution of very large (>500 kb) CNVs in BD compared with SZ, most notably for deletions >1 Mb (*P*=9 × 10^−4^).

## Introduction

There is strong evidence for a substantial genetic contribution to risk for both bipolar disorder (BD) and schizophrenia (SZ).^1^ Recent studies have provided evidence that while some alleles confer a specific susceptibility to either BD or SZ, others confer susceptibility to both disorders.^2^ Large-scale genome-wide association studies have identified common variants (minor allele frequency >0.01) that increase risk of both BD and SZ at *CACNA1C*, *ANK3*, *ITIH3-ITIH4*, *ZNF804A* and *NCAN.*^3, 4, 5, 6, 7, 8^ Polygenic risk score analyses have also shown that large numbers of alleles that are yet to be identified at genome-wide significance are over-represented in SZ cases and are also over-represented in cases of BD.^9^

Copy number variants (CNVs) are structural genomic variations that are >1 kb in size and occur in the form of deletions, duplications, insertions and inversions (reviewed in Malhotra and Sebat^10^). Large, rare CNVs (>100 kb in size and present in <1% of individuals) increase risk for SZ and other neurodevelopmental disorders including autism spectrum disorders, intellectual disability, attention deficit hyperactivity disorder, developmental delay and epilepsy.^10, 11, 12, 13, 14^ In a recent study, we showed that 11 CNV loci are significantly associated with SZ and four more loci are potentially implicated.^15^ Overall, 2.5% of individuals with SZ carry at least one of these 15 CNVs, compared with 0.9% of controls.^15^ CNVs have been less well studied in BD; the evidence for their involvement in typical forms of the disorder is less clear-cut than in SZ.^2^ Studies in general have not found a significant increase in the rate of rare CNVs in individuals with BD compared with controls.^16, 17, 18, 19^ However, it has been reported that singleton deletions over 100 kb in length are more frequent in BD cases,^17, 18^ that an increased rate of CNVs occurs in early-onset BD cases,^20^ that the frequency of *de novo* CNVs are significantly more frequent in BD cases (with an onset below 18 years of age) than controls^21^ and that the rate of *de novo* CNVs in BD are intermediate between SZ and controls.^22^ We have not observed such excesses in the Wellcome Trust Case Control Consortium (WTCCC) BD CNV analysis, and we did note that the overall rate of CNVs seen in BD cases was less than that found in individuals with SZ^16^ and significantly lower than in a reference group with other non-psychiatric disorders.^23^

In a recent review, Malhotra and Sebat^10^ reported the rate for CNVs at specific loci that have received the strongest support for a variety of disorders (intellectual disability, developmental delay, autism spectrum disorder, SZ, and BD and recurrent depression) by combining all available data. The CNV occurrence at each locus was reported for BD and recurrent depression combined, and suggested an increase of CNVs at four loci: deletions at 3q29 and 22q11.21, and duplications at 1q21.1 and 16p11.2. The incidence of CNVs at these loci for just BD was not reported separately.

In this study, we examined CNVs in a new United Kingdom BD data set (*n*=2591), the Bipolar Disorder Research Network sample (www.BDRN.org). This sample is independent of the WTCCC data set we previously reported.^16, 23^ We performed three types of analysis of rare CNVs (<1% of the population): (1) we compared the rate of CNVs in BD vs controls at 15 loci that received support for association with SZ, using all available large data sets.^15, 24^ (2) We examined each gene across the genome for exon-disrupting CNVs in BD compared with controls, using a size cutoff of 10 kb. (3) We compared the burden of very large CNVs (>500 kb in size) in BD with that in a large SZ data set from the United Kingdom (CLOZUK and CardiffCOGs, *n*=6882) and with publicly available data from control individuals typed on similar arrays (*n*=8842).^24^

## Materials and methods

### Bipolar disorder cases

The BD sample is a new collection called the Bipolar Disorder Research Network sample (www.BDRN.org), recruited in collaboration with the Stanley Center for Psychiatric Research at the Broad Institute of MIT and Harvard. All participants were unrelated, white European, living in the British Isles. The protocols and procedures were approved by the relevant ethics review panels where patients were recruited. The individuals were recruited if they suffered with a major mood disorder in which clinically significant episodes of elevated mood had occurred. Bipolar cases were excluded if they had experienced mood or psychotic illness only as a result of alcohol or substance dependence or medical illness or medication; or were biologically related to another study participant. The following methodology was used for assessment of bipolar cases: a semi-structured lifetime ever psychiatric interview (Schedules for Clinical Assessment in Neuropsychiatry),^25^ followed by clinical ratings and a best-estimate lifetime diagnosis according to the Research Diagnostic Criteria.^26^ In cases where there was doubt as to the best-estimate lifetime diagnosis, diagnostic and clinical ratings were made by at least two members of the research team blind to each other's ratings.

The BD cases consisted of 2637 individuals of which 30.8% were male. The mean age of recruitment was 46 years (s.d.=12), with a mean age at first impairment due to BD of 28 years (s.d.=11). There were 61% bipolar I disorder/mania, 32% bipolar II disorder/hypomania and 7% schizoaffective disorder, bipolar type.

### Schizophrenia cases

For comparison of BD with SZ, we used two UK-based samples that we previously described and analysed for CNVs.^12, 15, 24^ The CLOZUK SZ cases (*n*=6558) consist of individuals taking the antipsychotic clozapine. Subjects (71% male) were aged 18–90 years with a recorded diagnosis of treatment-resistant SZ according to the clozapine registration forms completed by their psychiatrists. The use of these anonymised samples for genetic association studies was approved by the local Ethics Committee. The CardiffCOGS (*n*=571) is a sample of clinically diagnosed SZ patients recruited from community, inpatient and voluntary sector mental health services in the United Kingdom. Interview with the SCAN instrument^25^ and case-note review was used to arrive at a best-estimate lifetime diagnosis according to DSM-IV criteria.^27^

### Controls

The control data sets comprised the same sample described in Rees *et al.*^24^ in a study of SZ, we did not include WTCCC1 controls used in Rees *et al.,*^24^ as they have been previously used in our WTCCC BD CNV analysis.^16^ The remaining controls (before quality control (QC) filtering) from Rees *et al.*^24^ were participants in a smoking cessation study from the United States (*n*=1491), individuals from the United States who took part in a study of melanoma (*n*=3102, ~2/3 individuals were affected with melanoma), individuals from Germany who were participants in a refractive error study (KORA study) (*n*=1869) and WTCCC2 controls (National Blood Donors cohort: *n*=1392 and the 1958 British birth cohort: *n*=1521). These data sets were chosen because they had been genotyped with Illumina (San Diego, CA, USA) arrays that have a high probe overlap with the arrays used to genotype the BD cases in the current study. Full details of these samples are available in the Supplementary Material. A total of 93.5% of controls were of European descent.

### Genotyping and QC filtering

Details of the arrays used for genotyping the data sets are available in the Supplementary Material; Supplementary Table S1. The steps for genotyping and QC filtering for the BD cases were performed as described in the previously reported SZ sample.^15^ To ensure that the CNV calling was comparable across the different arrays, only probes that were present on all arrays were analysed, resulting in a total of 520 766 probes. In addition, to avoid batch effects, raw intensity files from each BD, SZ and control data set were analysed independently.

The Illumina GenomeStudio software (v2011.1) was used to process the raw intensity data, generating Log R Ratios and B-allele frequencies. BD samples were excluded if any of the following QC statistics constituted an outlier within their source data set: Log R ratios s.d., B-allele frequency drift, wave factor and total number of CNVs. Out of the 2637 BD samples with array data, 46 were excluded due to (i) poor QC, (ii) duplicates or related (piHat>0.1) individuals of this or previous BD studies by identity by descent and (iii) incorrect gender. The numbers and the ethnicities of these subjects are listed in Supplementary Material; Supplementary Table S2. The final numbers after exclusions for QC and duplicates were 2591 BD cases, 6882 SZ cases and 8842 controls. These samples were used for the comparisons between SZ and BD, and for the identification of new loci, whereas larger, publicly available data sets were used for the analysis of the 15 SZ-associated loci.

CNVs went through QC steps described in more detail in the Supplementary Methods. Briefly, CNVs were included if their frequency was <1% (applied using PLINK^28^). Subsequently, CNVs were further validated by applying a median *Z*-score outlier method of validation.^29^ Different size cutoffs were used for the different types of analysis, as detailed below.

### Statistical analysis

The rates of CNVs at specific loci for BD and controls were compared using Fisher's exact test (two tailed). In order to determine genome-wide significance, we followed a previous practice by employing a Bonferroni correction for multiple testing of recurrent CNVs that are flanked by segmental duplications.^11, 15, 24, 30^ The resulting *P*-value threshold of 4.1 × 10^−4^ is based on 120 genomic regions that are prone to recurrent CNVs (*P*=0.05/120). When examining associations with CNVs at individual genes, accounting for testing ~20 000 genes, we employed a gene-wide association threshold of *P*<2.5 × 10^−6^.

The burden analysis of very large, rare CNVs was performed with PLINK^28^ using 10 000 permutations. The analysis was stratified by CNV size (500 kb–1 Mb and >1 Mb), testing deletions and duplications separately. To identify novel loci a gene-based approach was used.^24^ Each gene in the genome was examined for exon-disrupting CNVs using hg19 reference sequence gene coordinates (UCSC genome browser and includes non-coding RNAs). Deletions and duplications >10 kb were counted and analysed separately, and significance levels were generated using Fisher's exact test (two tailed) comparing the incidence in BD cases against controls. Potential regions were excluded if manual inspection of *Z*-scores, Log R ratios and B-allele frequency traces suggested that they were unreliable.^24, 29^

Power calculations were performed using the online open source genetic power calculator (http://pngu.mgh.harvard.edu/~purcell/gpc/).^31^ Frequencies and odd ratios for BD were taken from the review by Malhotra and Sebat.^10^

## Results

### Previously implicated SZ loci

We examined the rates of CNVs in BD cases and control groups at 15 loci previously implicated in SZ.^15^ The CNV rates were based on the following data sets: (i) BDRN cases in this current study, (ii) data from a meta-analysis by Malhotra and Sebat^10^ for BD cases (after excluding those with major depression), (iii) BD cases from the study by Bergen *et al.*,^18^ in a Swedish population and (iv) samples used in our previous CNV study^16^ using the BD cases from the WTCCC1 for loci that were not included in the Malhotra and Sebat^10^ study. In some instances, one or more of these four sources did not provide data for a particular CNV locus. A full description of which sources contributed to each of the 15 CNVs analysed is provided in the Supplementary Material; Supplementary Table S3. The CNV occurrence in controls are taken from Rees *et al.*,^15^ which reports the data from large combined control data sets ranging from 27 045 to 81 821 samples in total.

The rates of CNVs in all reported large BD data sets and control groups at the 15 loci are presented in Table 1. For 3 of the 15 CNVs, we noted nominally significantly higher rates in BD cases than in controls (without correction for multiple testing). The strongest evidence for association was obtained for duplications at 16p11.2, which were increased in BD cases (0.13%) compared with controls (0.03%) with a combined *P*-value of 2.3 × 10^−4^, surpassing our genome-wide significance threshold for this type of CNV (see Methods) and strengthening the evidence from previous studies. Supplementary Figures S1 and S2 indicate the positions of the duplications, Log R ratios and B-allele frequency traces using Illumina GenomeStudio in the three BD samples across 16p11.2. The other nominally significant loci were duplications at 1q21.1 and deletions at 3q29 (*P*-values, 0.022 and 0.03, respectively), which do not survive any multiple-testing correction.

### Analysis of novel CNV loci

Genes across the genome were examined for CNVs that disrupted exons in the BD BDRN sample set (*n*=2591) and control samples (*n*=8842), excluding the 15 previously implicated SZ loci reported in Table 1. A total of 55 genes mapping to 20 distinct genomic regions were enriched among our BD cases with nominal levels of significance (two-sided Fisher's exact test, *P*-value<0.05) (Supplementary Table S4), but no gene reached the genome-wide association threshold for the 20 000 genes examined (*P*<2.5 × 10^−6^). Within our data set, the strongest evidence for association was for duplications at *ATF7IP2 (*encoding activating transcription factor 7—interacting protein 2) located at 16p13.2–p13.13, which was found in eight BD cases (0.31%) and in four controls (0.045%, two-sided Fisher's exact test, *P*=1.4 × 10^−3^). The gene, *GRIN2A* encoding glutamate receptor, ionotropic, *N*-methyl-D-aspartate, subunit 2A, lies downstream of *ATF7IP2*. Its first exon is disrupted in seven BD cases and five controls (0.27% BD vs 0.057% controls, two-sided Fisher's exact test, *P*=8.1 × 10^−3^). Supplementary Figure S3 indicates the positions of the duplications in all samples across *ATF7IP2* and *GRIN2A*.

In addition, there is evidence of association for duplications at the gene *CGNL1* (cingulin-like 1) at 15q21.3, intersected in 13 BD cases (0.50%) and 19 controls (0.21%), two-sided Fisher's exact test, *P*=0.021. A significant excess of duplications at this gene have been previously observed in SZ.^24^ Supplementary Figure S4 indicates the positions of the duplications in the cases and controls across *CGNL1*.

### CNV burden analysis

We compared the rate of CNVs in BD with those in SZ cases and in controls. The burden analyses were performed only on the BD, SZ and control data sets where we had access to raw data and that were genotyped with Illumina arrays (BD: *n*=2591; SZ: *n*=6882; controls: *n*=8842), as described in the Materials and methods section. As CNV burden analysis is highly susceptible to technical bias, we limited it to the largest class of CNVs, those >500 kb, which should be called reliably on all arrays used in different data sets. BD cases were not significantly different from controls in any comparison (Table 2). SZ cases had more deletions >1 Mb compared with BD cases (two sided, *P*=9 × 10^−4^). Duplications in the size range of 500 kb–1 Mb were also more common in SZ compared with BD cases (two sided, *P*=0.045). As this excess could be due to the already implicated 15 SZ CNV loci (listed in Table 1), the analysis was repeated after removing CNVs in these loci. Neither of the comparisons remained even nominally significant, suggesting that a large part (but not all) of the excess in SZ is due to CNVs at those known loci: (deletions >1 Mb, SZ 0.87%, BD 0.54%, *P*=0.12; duplications, 500–1 Mb, SZ 5.1%, BD 4.3%, *P*=0.12).

## Discussion

It is well documented that specific CNVs contribute to the susceptibility of SZ, however, the involvement of CNVs in BD is less compelling. In a new independent BD sample, we have studied rare, large CNVs and analysed their frequencies in this sample and in previously reported BD, control and SZ data sets.^10, 15, 16, 18, 24^ The incidence of CNVs in this new BD data set had not been previously reported, although a large proportion of the sample has been used for genome-wide association studies.^3^ The SZ, BD and control samples were genotyped on a variety of Illumina arrays, but only probes present on all arrays were analysed (*n*=520 766). In addition, for all data sets (SZ, BD and controls) the methodology used was identical for CNV calling and statistical analysis.

Combining the BDRN BD data with recently reported CNV analyses^10, 15, 16, 18, 24^ provided support for three of the 15 previously implicated CNVs in SZ. Two of the loci, 1q21.1 duplication and 3q29 deletion were nominally significant with a two-sided *P*-value of 0.022 and 0.03, respectively, and these results do not survive correction for multiple testing. The strongest evidence was for duplications at 16p11.2 (two-sided *P*-value=2.3 × 10^−4^), which survives correction for the number of potential recurrent CNV loci in the genome (*P*-value<4.2 × 10^−4^) and is the most significant BD-associated CNV to date. The locus was implicated in BD before^10, 32^ (*P*=8 × 10^−4^ for BD and major depression combined,^10^ *P*=0.017 for BD^32^), but the current study raises the statistical support to above a level that is regarded as genome-wide significant for this type of CNV locus. Additional phenotypic details for the three carriers of 16p11.2 duplication are available in Supplementary Information. All three individuals had a DSM-IV rating of bipolar I disorder, and had episodes of depression as well. There was nothing unusual in their recorded presentations and at least two of them appeared to have no cognitive deficits, attaining O- or A-levels at school. The locus 16p11.2 has been also previously implicated in neuropsychiatric disorders via a genome-wide association study of mixed SZ and BD (psychosis) phenotypes, which revealed a novel variant at 16p11.2 showing genome-wide association for rs4583255 (*P*-value 6.6 × 10^−11^, odds ratio=1.08) located in the 593-kb CNV duplication region^33^ within the gene *TAOK2*. A significant excess of a combination of microdeletions and duplications at 16p11.2 has also been reported in major depressive disorder in a German sample.^34^

Our BD discovery sample consists of 2591 cases and 8842 controls, and has a power of 74 and 80% with an *α* of 0.05 to detect associations with duplications at 1q21.1 and 16p11.2, respectively. However, for the associations with the 15 SZ loci we used all available data sets, increasing the numbers of BD cases to ~4000–9000 and the numbers of controls to those that provided the definitive findings in SZ. Although the BD case numbers are still smaller than for SZ, it is clear (Table 1) that for some of the loci the frequencies in BD are very similar to those of controls, suggesting that this is not a power problem but more likely a genuine difference between SZ and BD.

We provide a list of the top hits for duplications and deletions at 55 genes that are more frequently affected in BD cases compared with controls (Supplementary Table S4). The significance for any of these genes does not survive a Bonferroni correction for multiple testing of 20 000 genes separately for deletions and duplications (*P*<2.5 × 10^−6^). The strongest evidence for association was for duplications at *ATF7IP2*. *GRIN2A*, a glutamate receptor, lies downstream of *ATF7IP2*, and although less significantly associated with BD, functionally it is the better candidate, as glutamate signalling pathways are thought to be involved in genetic predisposition to BD.^35^ In addition, *GRIN2A* is associated with SZ, meeting genome-wide significance.^36^ At the gene *CGNL1* (cingulin-like 1), there is overlap with our current BD data and SZ. Here, we report an excess of duplications in BD cases compared with controls in this gene, for which we also note an excess of duplications in SZ cases compared with controls.^24^ However, we note that the control samples used in the current BD study are not independent of those used in the previous SZ study. Replication in independent studies of both cases and controls is required to confirm the involvement of any of these loci with BD.

CNV burden analyses have shown an increased burden of large, rare CNVs in SZ: we reported a 2.5% higher rate of CNVs larger than 500 kb in SZ compared with controls.^24^ Using the same SZ data set to compare the burden of CNVs in BD revealed a significant difference between SZ and BD with respect to large, rare CNVs, in particular deletions. However, this excess was partially explained by the 15 loci already implicated in SZ. When comparing BD and control samples, we saw no significant difference in CNV burden for any of the CNV sizes examined. Both observations support our previous findings examining CNVs in BD cases and controls genotyped as part of the WTCCC study.^16^ These findings do not, however, exclude the involvement of CNVs in the susceptibility of BD at some specific loci. However, very large CNVs appear to contribute less to BD than to SZ.^16, 32^ Larger structural variants often appear to predispose to persistent, wide-ranging brain dysfunction, including those that affect cognitive and personality development.^2, 23, 37^

In summary, we have performed CNV analysis in a large independent BD data set and compared the CNV burden with both SZ and controls. Our data confirms previous findings, suggesting that there is a significant difference between SZ and BD in terms of CNV occurrence, in particular for large deletions >1 Mb. We do not rule out the possibility of CNV involvement in the susceptibility of BD at specific loci. In fact, we observe an increase of duplications at 16p11.2 and 1q21.1, deletions at 3q29 and the potential involvement of additional CNVs, all of which require replication.

## Figures and Tables

**Table 1:** Comparison of copy number variations (CNVs) in BD (BDRN data set and previously reported data for BD^10, 16, 18^), and the combined control data set^15^ at 15 SZ-CNV-implicated loci

		*CNV frequency, % (*n/N)				
*Locus*	*Position*	*Current BDRN BD study*	*BD cases combined (total)*	*Controls (total)*	*OR (95% CI)*	P*-value*
1q21.1 del	chr1:146 57–147 39	0.039% (1/2591)	0.033% (3/8968)	0.021% (17/81 821)	1.61 (0.47–5.50)	0.44
1q21.1 dup	chr1:146 57–147 39	0.039% (1/2591)	0.099% (8/8084)	0.037% (24/64 046)	2.64 (1.19–5.88)	0.022
*NRXN1* del	chr2:5015–5126	0% (0/2591)	0% (0/4288)	0.020% (10/51 161)	NA	1
3q29 del	chr3:195 73–197 34	0% (0/2591)	0.025% (2/8084)	0.0014% (1/69 965)	17.31 (1.57–190.97)	0.03
WBS dup	chr7:7274–7414	0% (0/2591)	0% (0/7250)	0.0058% (2/34 455)	NA	1
*VIPR2* dup	chr7:158 82–158 94	0% (0/2591)	0.043% (2/8084)	0.069% (17/24 812)	0.36 (0.08–1.56)	0.19
15q11.2 del	chr15:2280–2309	0.27% (7/2591)	0.17% (15/8966)	0.28% (227/81 802)	0.60 (0.36–1.02)	0.052
AS/PWS dup	chr15:2482–2843	0% (0/2591)	0% (0/8084)	0.0063% (3/47 686)	NA	1
15q13.3 del	chr15:3113–3248	0% (0/2591)	0.043% (2/8084)	0.019% (15/80 422)	1.32 (0.30–5.80)	0.66
16p13.11 dup	chr16:1551–1630	0.23% (6/2591)	0.11% (9/8084)	0.13% (93/69 289)	0.83 (0.42–1.64)	0.75
16p11.2 distal del	chr16:2882–2905	0% (0/2591)	0% (0/4288)	0.018% (5/27 045)	NA	1
16p11.2 dup	chr16:2964–3020	0.12% (3/2591)	0.13% (12/9129)	0.030% (19/63 068)	4.37 (2.12–9.00)	2.3 × 10^−4^
17p12 del	chr17:1416–1543	0.039% (1/2591)	0.049% (4/8132)	0.026% (17/65 402)	1.89 (0.64–5.63)	0.28
17q12 del	chr17:3481–3620	0% (0/2591)	0% (0/7250)	0.0054% (4/74 447)	NA	1
22q11.2 del	chr22:1902–2026	0% (0/2591)	0.012% (1/8084)	0% (0/77 055)	NA	0.095

Abbreviations: BD, bipolar disorder; CI, confidence interval; Del, deletions; Dup, duplications; NA, not applicable; OR, odds ratio.

Positions are in Mb for UCSC Build hg19.

**Table 2:** Burden analysis of large CNVs comparing SZ vs BD cases and BD cases vs controls (Con)

*Size range*	*CNV type*	*BD*	*SZ*	*Con*	P*-value, SZ v BD (% excess)*	P*-value, BD v Con (% excess)*
		*CNV freq (*n *CNV)*	*CNV freq (*n *CNV)*	*CNV freq (*n *CNV)*		
500 kb–1 Mb	Del	1.1% (29)	1.3% (90)	1.2% (102)	0.48	0.92
	Dup	4.4% (114)	5.5% (377)	4.5% (401)	0.045	0.79
>1 Mb	Del	0.62% (16)	1.5% (105)	0.61% (54)	9 × 10^−4^	1
	Dup	2.5% (65)	2.6% (180)	1.9% (168)	0.77	0.061

Abbreviations: CNV, copy number variations; BD, bipolar disorder; Del, deletions; Dup, duplications; SZ, schizophrenia.

CNVs are stratified by type (deletions and duplications) and size (500 kb–1 Mb and >1 Mb).
