# Supplementary material for: Copy number variation in bipolar disorder
Source: Mol Psychiatry. 2015 Jan 6;21(1):89–93. doi: 10.1038/mp.2014.174 (PMC5038134; doi:10.1038/mp.2014.174)
Supplement: Supplementary Information [file mp2014174x1.doc]

**Supplementary material**

1. Samples and Genotyping Information
2. Sample and CNV Quality Control
3. Incidence of CNVs at schizophrenia implicated loci in previous datasets.
4. Duplications at chromosome 16p11.2
5. Phenotype details of the 3 bipolar disorder individuals carrying the chromosome 16p11.2 duplications
6. Exon disrupting CNVs comparison in bipolar disorder cases and controls.
7. UCSC tracks of *GRIN2A* & *ATF7IP2* and *CGNL1*
8. **Samples and Genotyping Information**

Genotyping of the 7 129 schizophrenia (SZ) and 2 627 bipolar disorder (BD) patients was performed at the Stanley Centre for Psychiatric Research of MIT and Harvard on two separate arrays: Human OmniExpress-12v1 (Omni Express array), and HumanOmniExpressExome-8v1 (Combo array). The Combo array has SNPs from the Omni Express array and in addition those from the Illumina HumanExome-12v1_A (Exome array). Both the SZ and BD samples were genotyped in batches as indicated in Table S1.

The large control dataset was previously used in our CNV study of the 7 129 schizophrenia samples^1^. The control raw intensity files were obtained from the Database of Genotypes and Phenotypes (dbGAP) and had been genotyped on Illumina arrays with similar coverage to the arrays used to genotype both the SZ and BD cases. These controls had not been used in previous studies on BD for copy number variation analysis (CNV). The datasets obtained and used in this study are summarised in Table S1.

Table S1. Summary of case and control datasets prior to and after quality control filtering.

The ethnicities of the SZ and BD cases were provided by the psychiatrists and the ethnicities of the controls were available as supplementary notes along with the raw intensity data, usually based on self-reports. As previously shown^1,2^, the ethnicities for the SZ and controls were also derived from principle components analysis (PCA) combining the data with Hapmap genotypes, as was performed for the BD samples. All BD samples were of European descent. The non-European individuals in the SZ and control datasets were either grouped into African or ‘other’ as they constitute a very small proportion of the total sample^1^.

Table S2. Number of case and control samples pre and post quality control steps, divided by ethnicity.

1. **Sample and CNV Quality Control (QC)**

QC followed the methods used in our previous papers ^1,2^. All datasets, cases and controls were analysed independently to avoid batch effects. The raw intensity data was processed for each sample using Illumina Genome Studio software (v2-11.1). Each dataset had their own egt cluster file defined on their own data in order to generate accurate Log R ratios and B-allele frequencies for subsequent CNV detection. PennCNV^3^ was used to call CNVs from the data following the standard protocol and adjusting for GC content. A total of 5 different Illumina array platforms were used for genotyping all datasets, each with different SNP probe sets. In order that the CNV calling was comparable across the different arrays used, we analysed the 520 766 probes present on all arrays.

Sample level QC was performed using the QC metrics generated by PennCNV^3^. The QC for the SZ cases and controls had previously been performed^1,2^. Identical measures were used for the BD datasets, including Log R ratio (LRR) standard deviation, B-allele frequency drift, wave factor and total number of CNVs called per person. Samples were excluded if for any one of these metrics they constituted an outlier in their source dataset. The number of samples excluded from each dataset is shown in Table S2.

After all poorly performing or duplicated/related samples had been removed from the analysis, QC of the CNVs was performed. QC of the CNVs included joining together CNVs in the same individual if the distance separating them was less than 50% of their combined length. CNVs were excluded if they were <10kb, covered by < 10 probes, had a probe density > 1 probe/20kb, or had >50% of their length overlapped by low copy repeats (LCRs). A CNV locus frequency filter of 1% was applied using PLINK^4^, with a locus defined as CNVs that overlap by 50% of their length. Rare CNVs were subsequently required to pass a median Z-score outlier method of validation, as detailed in Kirov *et al*., (2012)^5^. A median Z-score value for all standardised probe intensities within a CNV was used to verify true deletions and duplications, which are represented as outliers in the median Z-score distribution. The Z-score method helps to remove false positive CNVs, and can also identify any missed CNVs. CNVs that had marginal Z-scores were inspected manually for LRR and B-allele frequency traces using Illumina GenomeStudio v2011.1.

1. **Incidence of CNVs at schizophrenia implicated loci in previous datasets.**

Table S3 shows the CNV frequencies for each dataset used in this combined analysis at the 15 SZ implicated loci.

Table S3. Copy Number Variation (CNV) frequencies in the BDRN BD and previously reported datasets and controls at 15 schizophrenia (SZ) implicated loci. From the Grozeva *et al*., study ^8^ we only took CNVs in NRXN1 and distal 16p11.2, as the other loci are included in the Malhotra & Sebat study ^6^.

del: deletion, dup: duplication, UP: recurrent unipolar depression. Positions are given in Mb for UCSC Build hg19.

1. **Duplications at chromosome 16p11.2**

Rigorous quality control, including the median Z-score method, as discussed in the methods and supplementary information, was performed for all relevant CNVs. Figures S1 displays the chromosomal locations of the 16p11.2 duplications in the 3 BD carriers and Figure S2 shows the LRR and BAF traces for this locus in the 3 BD 16p11.2 duplication carriers, thus demonstrating the presence of the duplications.

Figure S1. UCSC track of duplications at chromosome 16p11.2 carried by 3 individuals with bipolar disorder.

Figure S2. Log R Ratio and B-allele frequency at chromosome 16p11.2 (chr16:29,64-30,20) indicating duplications carried by 3 BD individuals. Sample 1 and 2 were genotyped on the Human OmniExpress-12v1 (Omni Express) array and Sample 3 using the Human OmniExpressExome-8v1 (Combo) array.

1. **Phenotype details of the 3 bipolar disorder individuals carrying the chromosome 16p11.2 duplications**
2. Individual 1

Female, aged 59 at interview. She had suffered from three episodes of mania and 3 episodes of depression during her lifetime. She had not attained any formal educational qualifications and had worked as a ‘service, shop or market worker’. No occurrence of postpartum psychosis (nor blues) despite having had children, no history of suicidal behaviour, alcohol abuse or dependency.

1. Individual 2

Female, aged 52 at interview, with lifetime mood congruent psychotic features. This individual was 21 years old when the first impairment due to affective/psychotic illness occurred. She suffered from 10 and 20+ episodes of mania and depression respectively during her lifetime. Has suffered from migraine. She has a positive family history affective disorder, with at least 3 members of the family with core or broad diagnosis. Attained O-level/GCSE’s, and has been employed as a ‘Professional’ (highest occupational achievement). Following childbirth, this individual has experienced mania within 6 weeks of delivery. Has had a ‘Suicide attempt unlikely to result in death’.

1. Individual 3

Male, aged 50 at interview, with lifetime mood congruent psychotic features: virtually all content congruent with the affective state. Heaviest alcohol problems resulted in psychiatric/psychological problems. This individual was 17 years old when the first impairment due to affective/psychotic illness occurred. He suffered from 10 and 10+ episodes of mania and depression respectively during his lifetime. Has suffered from migraine. He attained A-level/AS levels/Scottish Highers/HND/BTEC educational level and has been employed as a ‘Technician or associate professional’. He has a history of a serious suicidal attempt, and a diagnosis of alcohol abuse.

1. **Exon disrupting CNVs comparison in bipolar disorder cases and controls**

**Table S4.** CNV counts for all 55 genes that reached nominal levels of significance in the BD case sample. CHR: chromosome; Positions are in base pairs for UCSC Build hg19.

1. **UCSC tracks of *GRIN2A & ATF7IP2* and *CGNL1***

Location of the CNVs disrupting *GRIN2A* and *ATF7IP2*, and *CGNL1* associated with BD.

Figure S3. UCSC track of the duplications at *GRIN2A* and *ATF7IP2*.

Figure S4. UCSC track of the duplications at *CGNL1*.

**References**

1. Rees E, Walters JT, Georgieva L, Isles AR, Chambert KD, Richards AL *et al*. Analysis of copy number variations at 15 schizophrenia-associated loci. *Br J Psychiatry* 2014; 204: 108-114.
2. Rees E, Walters JT, Chambert KD, O'Dushlaine C, Szatkiewicz J, Richards AL, *et al*. CNV analysis in a large schizophrenia sample implicates deletions at 16p12.1 and SLC1A1 and duplications at 1p36.33 and CGNL1. *Hum Mol Genet* 2014; 23: 1669-1676.
3. Wang K, Li M, Hadley D, Liu R, Glessner J, Grant SF *et al*. PennCNV: an integrated hidden Markov model designed for high-resolution copy number variation detection in whole-genome SNP genotyping data. *Genome Res* 2007; 17: 1665-1674.
4. Purcell S, Neale B, Todd-Brown K, Thomas L, Ferreira MAR, Bender D *et al*. PLINK: A Tool Set for Whole-Genome Association and Population-Based Linkage Analyses. *Am J Hum Genet* 2007; 81: 559-575.
5. Kirov G, Pocklington AJ, Holmans P, Ivanov D, Ikeda M, Ruderfer D *et al*. De novo CNV analysis implicates specific abnormalities of postsynaptic signalling complexes in the pathogenesis of schizophrenia. *Mol Psychiatry* 2012; 17: 142
6. Malhotra D, Sebat J. CNVs: harbingers of a rare variant revolution in psychiatric genetics. *Cell* 2012; 148: 1223-1241.
7. Bergen SE, O'Dushlaine CT, Ripke S, Lee PH, Ruderfer DM, Akterin S *et al*. Genome-wide association study in a Swedish population yields support for greater CNV and MHC involvement in schizophrenia compared with bipolar disorder. *Mol Psychiatry* 2012; 17: 880-886.
8. Grozeva D, Kirov G, Ivanov D, Jones IR, Jones L, Green EK *et al*. Rare copy number variants: a point of rarity in genetic risk for bipolar disorder and schizophrenia. *Arch Gen Psychiatry* 2010; 67: 318-327.
