# Supplementary material for: Copy number variation in bipolar disorder
Source: Mol Psychiatry. 2015 Jan 6;21(1):89–93. doi: 10.1038/mp.2014.174 (PMC5038134; doi:10.1038/mp.2014.174)
Supplement: Supplementary Figure 1 [file mp2014174x6.doc]

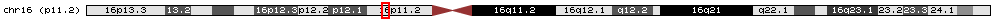


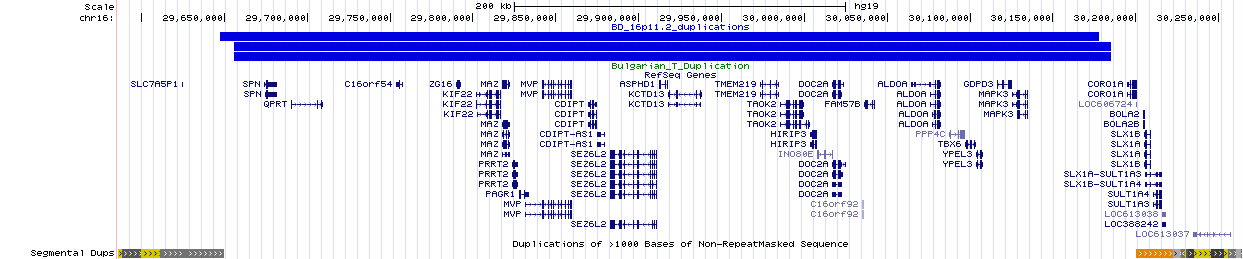


Figure S1. UCSC track of duplications at chromosome 16p11.2 carried by 3 individuals with bipolar disorder.
