# Supplementary material for: Copy number variation in bipolar disorder
Source: Mol Psychiatry. 2015 Jan 6;21(1):89–93. doi: 10.1038/mp.2014.174 (PMC5038134; doi:10.1038/mp.2014.174)
Supplement: Supplementary Figure 2 [file mp2014174x7.doc]

Samples 1-2

Sample 3

16p11.2 duplications

16p11.2 duplications

Figure S2. Log R Ratio and B-allele frequency at chromosome 16p11.2 (chr16:29,64-30,20) indicating duplications carried by 3 BD individuals. Sample 1 and 2 were genotyped on the Human OmniExpress-12v1 (Omni Express) array and Sample 3 using the Human OmniExpressExome-8v1 (Combo) array.
