# Supplementary figures and images for: Copy number variation in bipolar disorder
Source: Mol Psychiatry. 2015 Jan 6;21(1):89–93. doi: 10.1038/mp.2014.174 (PMC5038134; doi:10.1038/mp.2014.174)

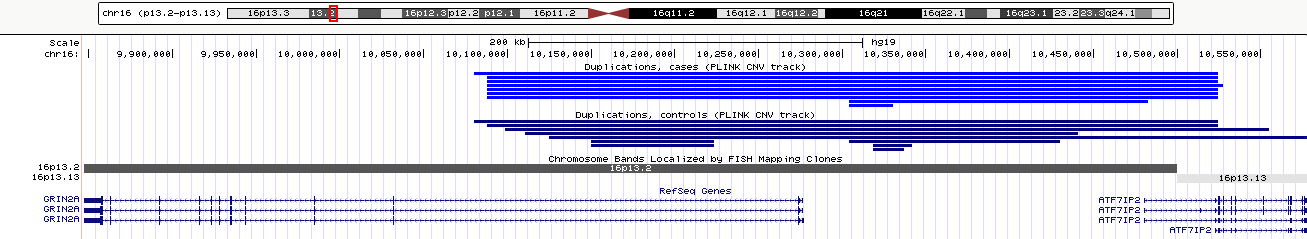


Figure S3. UCSC track of the duplications at *GRIN2A* and *ATF7IP2*.

Supplement: Supplementary Figure 3 [file mp2014174x8.doc]

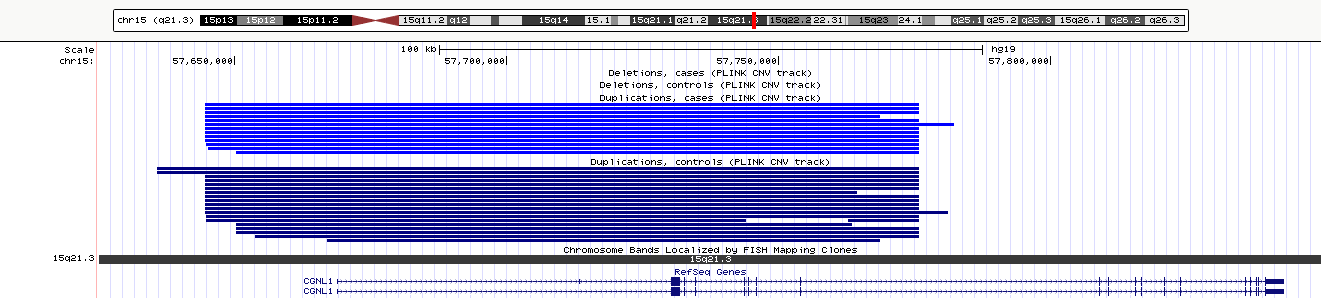


Figure S4: UCSC track of the duplications at *CGNL1*.

Supplement: Supplementary Figure 4 [file mp2014174x9.doc]
